# Changes in Gut Microbiota and Systemic Inflammation after Synbiotic Supplementation in Patients with Systemic Lupus Erythematosus: A Randomized, Double-Blind, Placebo-Controlled Trial

**DOI:** 10.3390/cells11213419

**Published:** 2022-10-29

**Authors:** Alvina Widhani, Samsuridjal Djauzi, Franciscus Dhyanagiri Suyatna, Beti Ernawati Dewi

**Affiliations:** 1 Allergy and Clinical Immunology Division, Department of Internal Medicine, Faculty of Medicine, Universitas Indonesia, Jakarta 10430, Indonesia; 2 Dr. Cipto Mangunkusumo Hospital, Jakarta 10430, Indonesia; 3 Doctoral Program in Biomedical Science, Faculty of Medicine, Universitas Indonesia, Jakarta 10430, Indonesia; 4 Department of Pharmacology and Therapeutics, Faculty of Medicine, Universitas Indonesia, Jakarta 10430, Indonesia; 5 Department of Microbiology, Faculty of Medicine, Universitas Indonesia, Jakarta 10430, Indonesia

**Keywords:** systemic lupus erythematosus, inflammation, synbiotic, probiotic, microbiota

## Abstract

Gut dysbiosis has a role in the pathogenesis of lupus. Synbiotic supplementation may restore the balance of gut microbiota. This study investigated whether synbiotics could improve gut microbiota and systemic inflammation in lupus patients. This randomized, double-blind, placebo-controlled trial was conducted in adult systemic lupus erythematosus (SLE) patients. Subjects were randomized to receive either synbiotics or a placebo. Fecal microbiota, hs-CRP, IL-6, and IL-17 were measured at baseline and after 60 days. Patients who fulfilled the inclusion criteria were randomized into synbiotic (n = 23) and placebo groups (n = 23). In the synbiotic group, hs-CRP was not significantly increased (1.8 [0.9; 4.85] vs. 2.1 [0.9; 4.25] mg/L; pre vs. post; *p* = 0.23), whereas in the placebo group hs-CRP was increased significantly (1.75 [0.4; 4.45] vs. 3.75 [0.58; 7.05] mg/L; pre vs. post; *p* = 0.005). In the synbiotic group, IL-6 decreased significantly (8.76 [6.62; 11.39] vs. 6.59 [4.96; 8.01]; pre vs. post; *p* = 0.02), while there was no significant change in IL-17 level. In the placebo group, there was no significant change in IL-6 and IL-17. Synbiotic supplementation increased the *Firmicutes:Bacteroidetes* ratio (0.05 ± 0.60 vs. −0.08 ± 0.63, synbiotic vs. placebo *p* = 0.48) and butyrate metabolism (*p* = 0.037) and decreased amino sugar and nucleotide sugar metabolism (*p* = 0.040). There was improvement in the SLE disease activity index 2K (SLEDAI-2K) score in the synbiotic group (14 [9; 16] vs. 8 [2; 12]; pre vs. post; *p* < 0.001), while no change in the placebo group (9 [8; 18.25] vs. 9 [5.5; 15]; pre vs. post; *p* = 0.31). Synbiotic supplementation could reduce systemic inflammation and SLE disease activity and alter the composition and functions of gut microbiota.

## 1. Introduction

Systemic lupus erythematosus (SLE) is a chronic multiorgan autoimmune disease with high morbidity. The interaction of genetic predisposition and environmental factors causes a loss of self-tolerance, which leads to the development of SLE [1]. Gut dysbiosis may contribute to the pathogenesis of SLE [2].

Gut microbiota participates in developing and balancing mucosal and systemic immunity to develop tolerance to harmless bacteria while providing an adequate response to pathogens [3]. Gut microbiota also affects intestinal permeability [4]. Increased intestinal permeability caused by the malfunction of tight junction proteins such as zonulin can increase the plasma levels of lipopolysaccharides and eventually cause low-grade inflammation [5]. The role of gut microbiota and their metabolites in SLE pathogenesis is probably mediated through several mechanisms, namely: molecular mimicry with self-antigens, bystander activation, epitope spreading, and bacterial translocation. These conditions lead to loss of self-tolerance, production of autoantibodies, attack of own cells, and chronic inflammation [6,7].

A study in young, female, lupus-prone mice showed depletion of *Lactobacilli* compared with age-matched healthy controls [8]. Studies in patients with SLE have reported significant decreases in the species richness, species diversity and *Firmicutes/Bacteroidetes* ratio compared with healthy controls and also changes in the metabolic function of gut microbiota [9,10,11,12]. Another study from China found increased *Bacteroidetes*, *Proteobacteria, Actinobacteria* and decreased Firmicutes. This study also revealed nine genera related to SLE: enrichment of *Klebsiella, Rhodococcus, Prevotella, Flavonifractor, Eubacterium, Eggerthella,* and *Incertae sedis* and depletion of *Pseudobutyrivibrio* and *Dialister* [13,14]. In SLE patients with high disease activity, the species richness and diversity severely decrease [6]. Studies have reported that disease activity is negatively correlated with *Bifidobacterium, Firmicutes*, *Lactobacillus*, and *Firmicutes/Bacteroidetes* ratio [6,9]. Meanwhile, it is positively correlated with *Ruminococcus gnavus, Streptococcus, Campylobacter, Streptococcus anginosus* and *Veillonella dispar* [6,7]. Increases in *Ruminococcus gnavus* in the stools of patients with lupus nephritis have been observed [7].

Synbiotic supplementation, a combination of prebiotics and probiotics which act synergistically, may restore the balance of gut microbiota, immune tolerance and immune homeostasis [6,15]. Studies have been conducted in a mice model of lupus with probiotics containing *Lactobacillus* and/or *Bifidobacteria* [6]. A study by Mu et al. used a mixture of five Lactobacillus strains (*Lactobacillus rhamnosus*, *Lactobacillus oris*, *Lactobacillus reuteri*, *Lactobacillus gasseri,* and *Lactobacillus johnsonii*) and showed that the mixture had an anti-inflammatory effect by increasing IL-10 and decreasing IL-6 [16]. Improvement of gut dysbiosis in patients with SLE might reduce systemic inflammation. There is no published clinical trial study about the effect of synbiotic on the inflammatory markers of SLE patients [6]. Studies in patients with inflammatory bowel disease and arthritis reported that there was a significant effect of probiotics on CRP reduction, while no effect was seen on IL-6 level [17]. We therefore investigated the effects of synbiotic supplementation on the composition and function of gut microbiota, high sensitivity C-reactive protein (hs-CRP), interleukin-6 (IL-6), and IL-17 in patients with SLE.

## 2. Materials and Methods

### 2.1. Study Design

This randomized, double-blind, placebo-controlled trial was performed at Cipto Mangunkusumo Hospital, Faculty of Medicine, Universitas Indonesia, Jakarta, Indonesia, with approval from the Research Ethics Committee of the Faculty of Medicine, University of Indonesia (registration ID: 804/UN2.F1/ETIK/2017). All methods were conducted in accordance with approved guidelines. Written informed consent was obtained from each patient before enrollment in the study. The study was registered retrospectively at www.clinicaltrials.gov, identifier NCT03494036. 

The calculated sample size was 23 subjects for each group, with 90% power and α = 0.05. Randomization was performed using a computer-based allocation system with blocking. A research assistant not involved in measuring the outcomes generated the allocation sequence before the study. The investigator enrolled the participants, and a research assistant assigned the participants to their respective groups. The participants, physicians, and laboratory staff had no knowledge of participant group allocations for the duration of the trial. The treatment assignment was not revealed until data collection had been completed

### 2.2. Subjects of the Study

The inclusion criteria were patients with SLE and gastrointestinal symptoms (abdominal pain, constipation, diarrhoea, or bloating) between 18 and 60 years old. Patients were excluded from the study if they had any of the following conditions at registration: (1) pregnancy or breastfeeding; (2) acute infection; (3) taking antibiotic treatment; (4) had consumed yogurt or taken probiotic supplementation in the 3 weeks prior to recruitment; or (5) taking corticosteroids (more than 20 mg prednisone or equivalent per day).

Patients who met the inclusion criteria and provided informed consent were randomized (1:1) into two groups: (1) a synbiotic-supplemented group who consumed synbiotic capsules containing 3 × 10^9^ colony forming units (CFU) of *Lactobacillus helveticus* R0052 60%, *Bifidobacterium infantis* R0033 20%, *Bifidobacterium bifidum* R0071 20%, and 80 mg fructo-oligosaccharides (Institute Rosell Inc., Montréal, Canada); or (2) the placebo group who were given capsules (identical in size, shape, and color) containing *Saccharum lactis.* Patients consumed the synbiotic or placebo tablets once daily for 60 days. All capsules were prepared before the study by pharmacists who were not otherwise involved.

All participants underwent identical processes during the trial. The primary goal was to evaluate changes in the composition and function of gut microbiota. The secondary goals were to evaluate changes in serum hs-CRP, IL-6, and IL-17. During the study period, patients were permitted to continue current medications but were prohibited from consuming other probiotic or synbiotic agents. Participants were considered dropouts if either of the following situations happened during the study: (1) yogurt was consumed or additional probiotic supplementation was taken more than once per week, or (2) changes were made to their prescribed steroid-sparing agent. Baseline data were collected, including: age, SLE disease activity index 2K (SLEDAI-2K) score, physician general assessment score, steroid dose, other medications (steroid-sparing agents, vitamin D, proton pump inhibitors, and statins), dietary composition, and body mass index. A dietary assessment was performed at each visit (at enrollment and weeks 4 and 8) by a certified nutritionist. Food frequency questionnaires and 24-h food recall were also used.

#### Assessment of Outcome

Serum hs-CRP, IL-6, and IL-17 levels were measured using enzyme-linked immunosorbent assay (ELISA) kits (DRG International Inc., Mountainside, NJ, USA; Legend MAX^TM^, Biolegend Inc, San Diego, CA, USA; and R&D system, Minneapolis, MN, USA; respectively), according to the manufacturer’s instructions.

Patients received explanations and written instructions on how to collect stool samples. Stool nucleic acid collection tubes (Norgen, Biotec Corp., Thorold, ON, Canada) were used and fecal samples were stored at −80 °C until DNA extraction was performed using QIAamp DNA stool mini kits (QIAGEN, Hilden, Germany), according to the manufacturer’s instructions. DNA purity and concentration were measured using a NanoDrop™ 2000 Spectrophotometer (Thermo Fisher Scientific, Waltham, MA, USA) and a Qubit Fluorometer (Invitrogen Life Technologies, Carlsbad, CA, USA). Before amplification, DNA with a purity ratio of 1.8 to 2.0 was diluted to 5 ng/μL. Polymerase chain reactions were performed using forward (5′–TCGTCGGCAGCGTCAGATGTGTATAAGAGACAGCCTACGGGNGGCWGCAG–3′) and reverse (5′–GTCTCGTGGGCTCGGAGATGTGTATAAGAGACAGGACTACHVGGGTATCTAATCC–3′) primers specific for 16S rRNA. The amplicons were purified following standard procedures, then quantified, pooled, and sequenced using MiSeq Reagent Kits (Illumina, San Diego, CA, USA), according to the manufacturer’s instructions.

After sequencing, the reads were assembled using FLASH (v1.2.7, http://ccb.jhu.edu/software/FLASH/ (accessed on 20 May 2018), which merged paired-end reads with sequence overlap longer than 20 bp. After assembly, the data were analyzed on the USEARCH pipeline (https://www.drive5.com/usearch/ (accessed on 20 May 2018) using default parameters. The primer sequences were truncated, and the reads were filtered based on expected error values. Only reads with expected error values <1.0 were used in this analysis. Unique reads and their abundance values were generated using the ‘fastx_uniques’ command in USEARCH. Operational taxonomic unit (OTU) clustering and chimera removal were performed using a UPARSE algorithm to produce OTUs with >97% similarity. The taxonomic affiliation of each OTU was predicted with USEARCH against the Ribosomal Database Project training set v16. Alpha diversity (richness, Chao1, and Shannon indexes) and beta diversity (unweighted UniFrac) were performed in USEARCH using OTU tables normalized to 10,000 reads. All data visualizations were performed using the R statistical package (https://www.r-project.org (accessed on 20 May 2018).

The top 50 OTUs were blasted against the Greengenes v13.5 database to obtain a Greengenes ID before the functional analysis using PICRUSt software (http://picrust.github.io/picrust/ (accessed on 20 May 2018) was completed. Potential changes in the microbiome at the functional level were determined using PICRUSt, with default settings, and the Kyoto Encyclopedia of Genes and Genomes database release 70.0. Changes were then visualized using the Statistical Analysis of Metagenomic Profile package (http://kiwi.cs.dal.ca/Software/STAMP (accessed on 20 May 2018). Bonferroni-corrected *p*-values <0.05 were used to determine the statistical significance of all analyses.

### 2.3. Statistical Analysis

Statistical analyses were performed using IBM SPSS statistics (version 20.0.0; IBM Corp., Armonk, NY, USA). Data were expressed as mean ± standard deviation (for normally distributed data) and median (interquartile range for data with skewed distributions). If data were distributed normally, comparisons of the results before and after synbiotic administration in each group and between the two groups were analyzed using paired or independent *t*-tests. Otherwise, the comparisons were analyzed using the non-parametric Wilcoxon rank-sum test and the Mann–Whitney *U* test. *p*-values <0.05 were considered statistically significant.

## 3. Results

### 3.1. Baseline Characteristics

**Figure 1:**
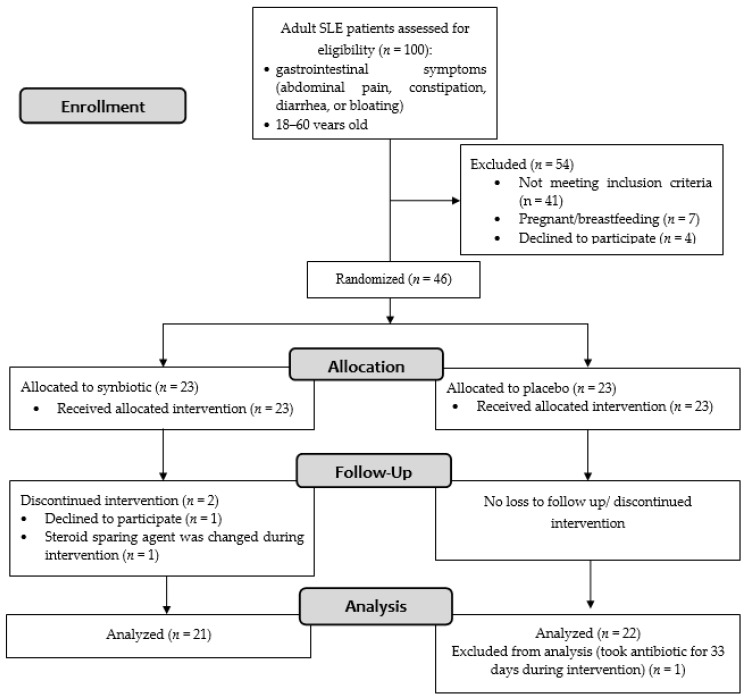
Flowchart diagram of the study.

Of the 100 patients screened, 46 patients fulfilled the inclusion criteria (Figure 1). Of the 46 female patients with SLE recruited in this study, 23 were randomly assigned to the synbiotic group and 23 to the control group. Recruitment ended after the required sample size was met. The patients’ baseline characteristics are summarized in Table 1. No significant differences were observed between groups concerning age, SLEDAI-2K score, physician general assessment score, the proportion of lupus nephritis, steroid dose, other medications (steroid-sparing agents, vitamin D, proton pump inhibitors, and statins), dietary composition, body mass index, and hs-CRP.

### 3.2. Follow-up and Adverse Events

In the synbiotic group, 21 patients completed the intervention: one patient withdrew consent, and another dropped out because of a treatment change. In the placebo group, all 23 patients completed the intervention, but one was excluded from the final analysis because she needed antibiotic treatment for 33 days.

One patient in the synbiotic group and one in the placebo group complained of abdominal pain at the beginning of the study. These symptoms resolved in 3 days without discontinuing intervention. There were two cases of hospitalization in the synbiotic group: one for a traffic accident and another for anemia. In the placebo group, one patient was hospitalized for thrombocytopenia, and one came to the emergency unit because of dyspnea. No cases of hospitalization were attributable to the trial interventions. No deaths occurred during the trial. Two patients in the synbiotic group and four in the placebo group had compliances below 100%.

We needed to increase the steroid dose toward the end of the intervention in eight patients from the placebo group and three patients from the synbiotic group because of clinical conditions (without knowing which group the subjects belonged to). Although the steroid dose was increased incrementally, it remained below 20 mg of prednisone or equivalent per day. 

### 3.3. Dietary Assessment

Changes in the daily intake of dietary energy, fiber, polyunsaturated fatty acids, vitamin A, vitamin E, vitamin C, and zinc were determined using 24 h food recall and food frequency questionnaires. Changes in dietary components were comparable between the synbiotic-supplemented and the placebo groups even though there were significant decreases in energy, fiber, vitamin E, vitamin C, and zinc intake in each group (Table 2). Analysis of the changes in energy and zinc intake was performed using an independent T-test because the data were normally distributed. Meanwhile, other changes in intake were performed using non-parametric tests because they were not normally distributed.

### 3.4. Changes in Serum Hs-CRP, IL-6, and IL-17

**Figure 2:**
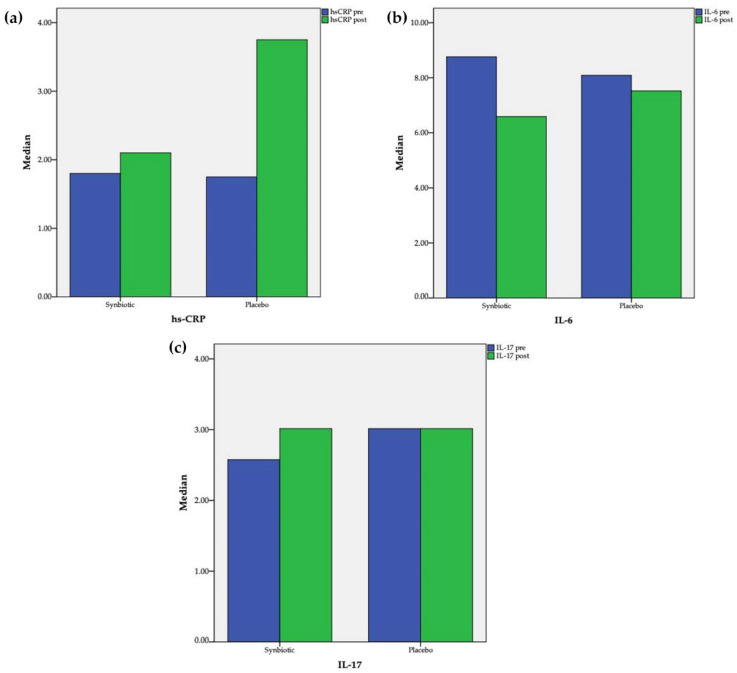
Changes after intervention in the synbiotic and placebo groups: (**a**) hs-CR; (**b**) IL-6; (**c**) IL-17.

After 60 days of supplementation, a significant increase in hs-CRP (1.75 [0.4; 4.45] vs. 3.75 [0.58; 7.05] mg/L, pre vs. post, *p* = 0.005) in the placebo group was observed and an insignificant change (1.8 [0.9; 4.8]) vs. 2.1 [0.9; 4.25] mg/L, pre vs. post, *p* = 0.23) in the synbiotic group was observed (Figure 2). Comparison between the two groups revealed that the changes of hs-CRP differed significantly (1.7 [−0.05; 5.13] vs. −0.2 [−0.7; 0.2], placebo vs. synbiotic, *p* = 0.002). Serum IL-6 decreased significantly in the synbiotic group (8.76 [6.62; 11.39] vs. 6.59 [4.96; 8.01] pg/mL; pre vs. post; *p* = 0.02); meanwhile in the placebo group, the level also decreased but not as significantly (8.09 [4.92; 18.38] vs. 7.52 [5.33; 18.3] pg/mL; pre vs. post; *p* = 0.78). Comparison between the two groups also revealed that the changes of IL-6 did not differ significantly (0.19 [−6.46; 3.69] vs. −2.19 [−4.32; 0.13], placebo vs. synbiotic, *p* = 0.27). The Spearman rho correlation test showed that there was a significant correlation between hs-CRP and IL-6 (r = 0.52; *p* < 0.001). Serum IL-17 did not change significantly in the synbiotic (2.58 [2.58; 3.24] vs. 3.01 [2.58; 3.24] pg/mL; pre vs. post; *p* = 0.90) or placebo groups (3.01 [2.58; 3.57] vs. 3.01 [2.58; 3.96] pg/mL; pre vs. post; *p* = 0.50). Comparison between the two groups further revealed that the changes of IL-17 also did not differ significantly (0 [−0.47; 0.29] vs. 0 [−0.44; 0.44], placebo vs. synbiotic, *p* = 0.60).

### 3.5. Gut Microbiota

**Figure 3:**
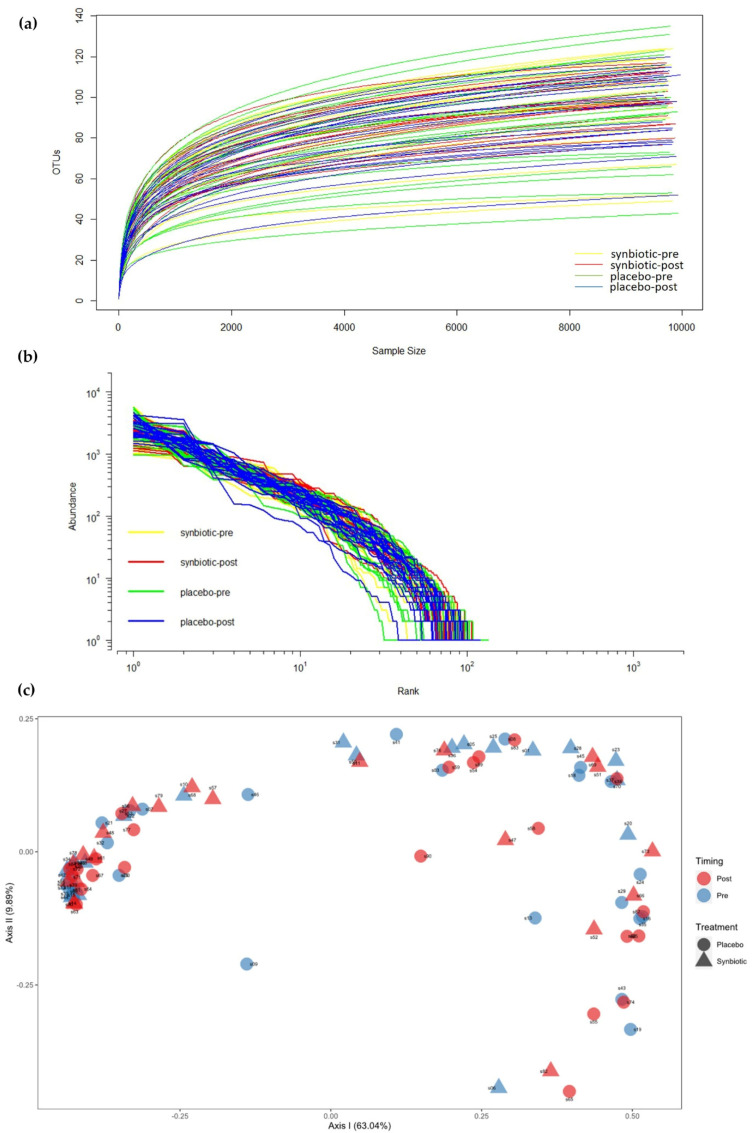
(**a**) Rarefaction curves from the synbiotic treatment group and the placebo group; (**b**) Rank abundance curve of the 16s rRNA gene; (**c**) Principal coordinate analysis (PCoA) comparing the four groups.

We analyzed 16s rRNA microbiome data from 89 fecal samples of the 46 patients (Details of content, length, and number of sequences for each fecal sample can be seen in the Supplementary File, Table S1). Paired-end sequencing of the amplicon targeting the V3–V4 region of the 16S rRNA gene generated 40,448 to 537,205 sequencing reads. Each paired-end read was joined to produce 36,760 to 492,913 reads. We then performed quality filtering of joined paired sequencing reads, which generated 19,569 to 251,043 sequencing reads. Rarefaction curves were similar between the synbiotic-supplemented and the placebo groups (Figure 3a). The rarefaction curves showed a trend toward plateauing, indicating that sequencing depths were sufficient to represent most microbial species. Rank abundance curves representing species abundance distributions are shown in Figure 3b. Most samples from the synbiotic and placebo groups had similar distributions. Few pre- and post-intervention samples from the placebo group and pre-intervention samples in the synbiotic group showed different distributions. Principal coordinate analysis (PCoA) is displayed in Figure 3c.

**Figure 4:**
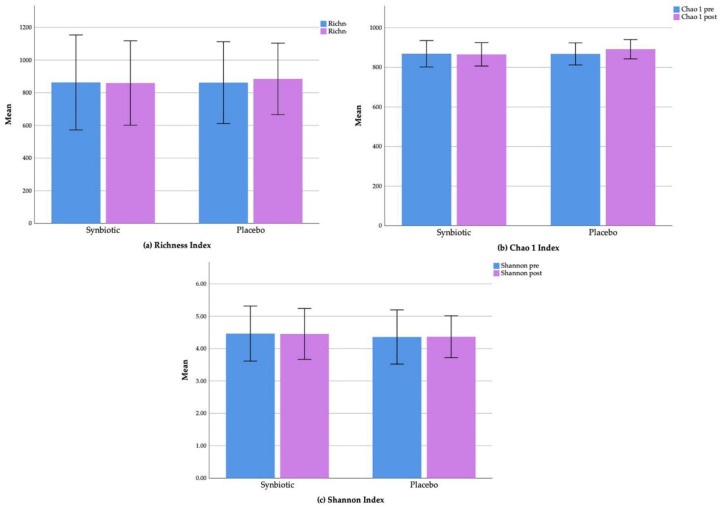
Changes of alpha diversity parameters: (**a**) Richness index; (**b**) Chao 1 index; and (**c**) Shannon index in the synbiotic and placebo groups.

We found no significant changes regarding the alpha diversity parameters (richness, Chao 1, and Shannon index) in either the synbiotic or the placebo groups. The mean richness indexes pre- and post-intervention for the synbiotic group were 862.90 ± 145.34 (mean ± standard deviation) and 859.52 ± 129.32, respectively (*p* = 0.92), and the indexes for the placebo group were 861.68 ± 125.20 and 884.77 ± 109.38, respectively (*p* = 0.41) (Figure 4a). Mean Chao 1 indexes (pre- and post-intervention) were 868.75 ± 146.55 and 865.63 ± 129.27 (*p* = 0.93) for the synbiotic group and 867.87 ± 125.95 and 891.65 ± 109.87 (*p* = 0.40) for the placebo group (Figure 4b). The mean Shannon indexes pre- and post-intervention for the synbiotic and placebo groups were 6.44 ± 0.61 and 6.42 ± 0.57 (*p* = 0.91) and 6.29 ± 0.60 and 6.30 ± 0.46, respectively (*p* = 0.93) (Figure 4c).

The proportion of *Bacteroidetes* was 57% (IQR 50.6%; 64.9%) and 49.3% (IQR 38.7%; 60.6%) in the synbiotic and placebo groups (*p* = 0.15), respectively. The proportion of *Firmicutes* was 31.16% (IQR 22.41%; 38.48%) in the synbiotic group and 30.36% (IQR 22.55%; 40.21%) in the placebo group (*p* = 0.96). The proportion of *Proteobacteria* was 6.89% (IQR 5%; 12.08%) in the synbiotic group and 8.36% (IQR 5.41%; 20.73%) in the placebo group (*p* = 0.28). The proportion of *Actinobacteria* was 0.64% (IQR 0.36%; 1.32%) in the synbiotic group and 0.59% (IQR 0.30%; 0.98%) in the placebo groups (*p* = 0.49). 

At the phylum level, only *Bacteroidetes* showed a significant relationship with disease activity (Table 3). Mean *Bacteroidetes* among SLE patients with mild disease activity (SLEDAI-2K score <6) was significantly lower than patients with moderate or high disease activity (SLEDAI-2K score ≥6) (43.19 ± 11.45 vs. 54.22 ± 13.81, *p* = 0.04, mild vs. moderate/high, respectively). The *Firmicutes/Bacteroidetes* ratio was higher among SLE patients with mild disease activity compared to patients with moderate/high disease activity. *Campylobacter* was significantly higher in patients with lupus nephritis. *Ruminococcus gnavus* was also higher in patients with lupus nephritis, but the result was not statistically significant. 

**Figure 5:**
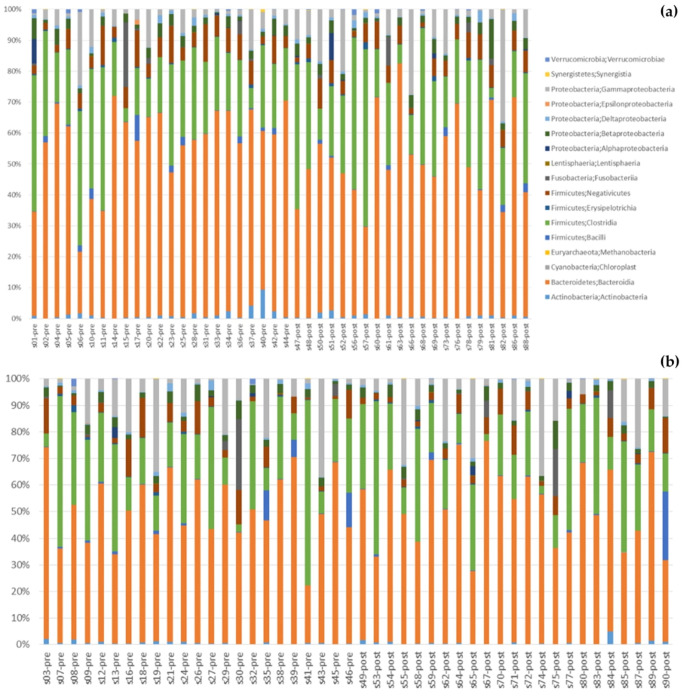

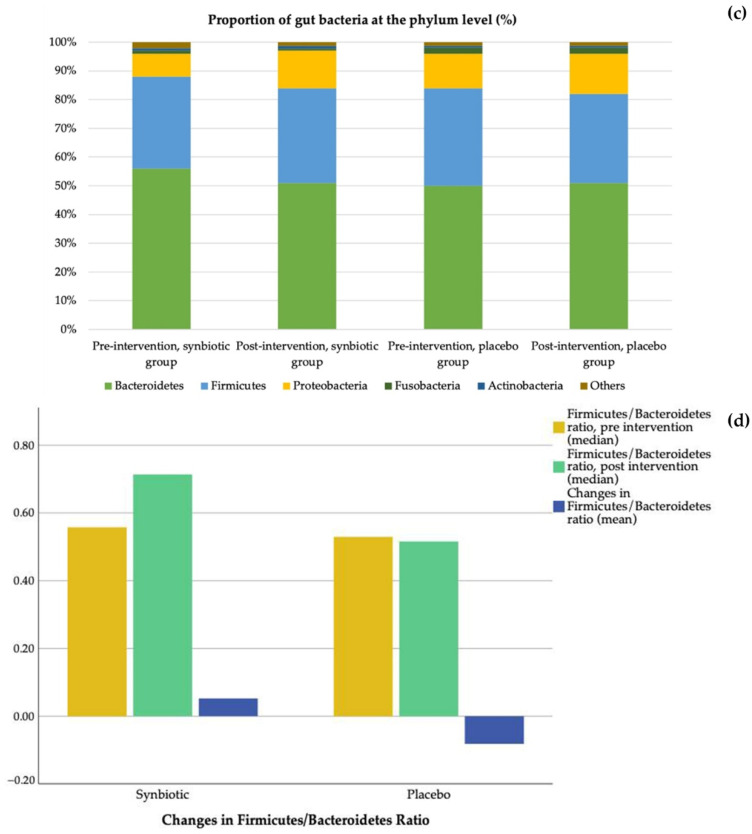
Changes in gut bacteria: (**a**) Class changes in the synbiotic group; (**b**) Class changes in the placebo group; (**c**) Comparison between the synbiotic and placebo group at the phylum level, before and after intervention; (**d**) Changes in *Firmicutes/Bacteroidetes ratio*.

Figure 5 shows changes in gut bacteria after the intervention. Changes at the class level can be seen in Figure 5a (synbiotic group) and Figure 5b (placebo group). At the phylum level (Figure 5c), after 60 days the proportion of *Bacteroidetes* decreased in the synbiotic group (56% to 51%) and no change in the placebo group (50% to 51%); this finding contrasted with the proportion of *Firmicutes*, which did not change in the synbiotic group (32% to 33%) and decreased in the placebo group (34% to 31%). The proportions of *Proteobacteria* increased in both groups after 60 days of intervention (8% to 13% and 12% to 14%) in the synbiotic and placebo groups, respectively. In the synbiotic group, the proportion of *Fusobacteria* and *Actinobacteria* did not change at all (1% to 0.8% and 0.9% to 0.9%, respectively). The proportions of *Fusobacteria* and *Actinobacteria* also did not change in the placebo group (2% and 0.7%, respectively). The median *Firmicutes/Bacteroidetes* ratio increased from 0.56 to 0.71 in the synbiotic group and decreased from 0.53 to 0.52 in the placebo group. Meanwhile, the *Firmicutes/Bacteroidetes* ratio increased (0.05 ± 0.60) in the synbiotic group and decreased (–0.08 ± 0.63) in the placebo group, but the difference was not significant (*p* = 0.48) (Figure 5d). 

**Figure 6:**
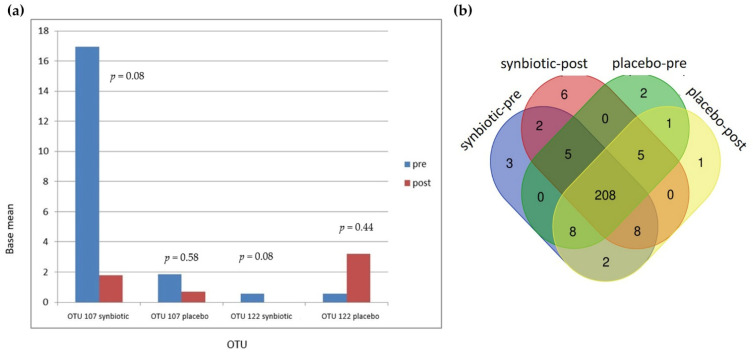

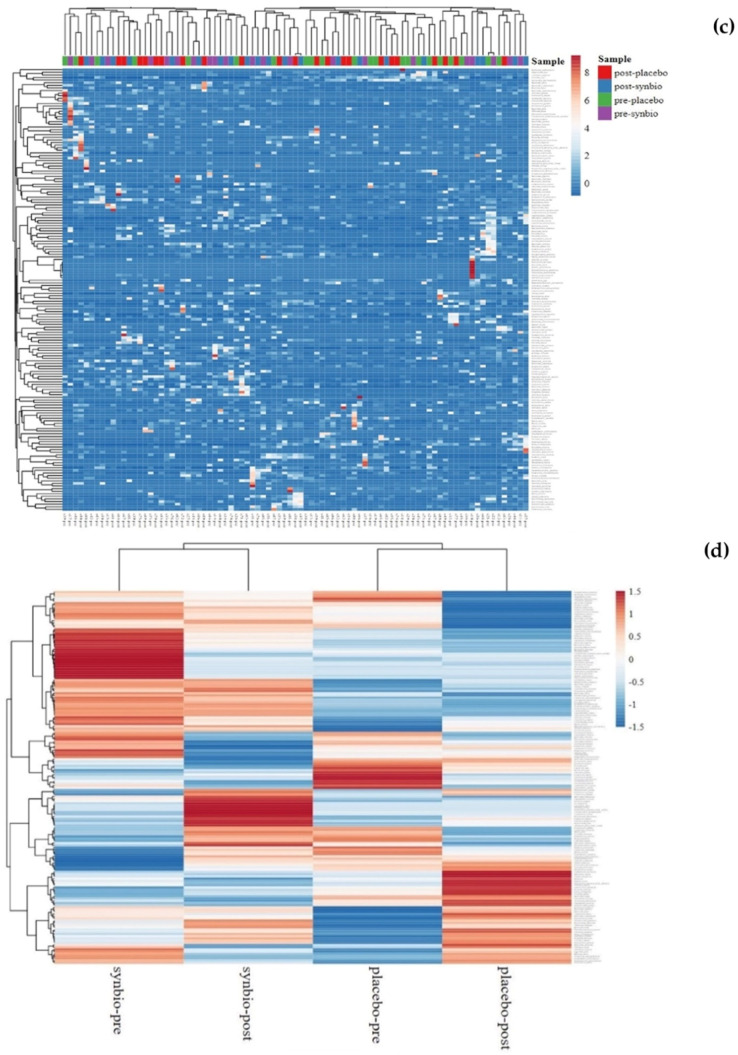
Changes in operational taxonomic units (OTUs): (**a**) Changes in OTU 107 and OTU 122; (**b**) Venn diagram showing the numbers of shared OTUs between groups; (**c**) Changes after intervention in both groups by heat map; (**d**) Average changes after intervention.

There were 251 OTUs identified in these samples (see Supplementary File Table S2). In the synbiotic group, there were two OTUs, OTU 107 (*Bacteroides salyersiae*) and OTU 122 (*Dialister invisus*), that showed a strong trend of changes in the base mean (Figure 6a). In the synbiotic group, OTU 107 and OTU 22 decreased from 16.95 to 1.78 (*p* = 0.08) and 0.55 to 0 (*p* = 0.08), respectively. In the placebo group, OTU 107 decreased from 1.84 to 0.70 (*p* = 0.58) while OTU 122 increased from 0.57 to 3.19 (*p* = 0.44). OTU 165 (*Bifidobacterium bifidum*), one of the bacteria in the synbiotic used in this study, increased in the synbiotic group from 1.04 to 1.21 (*p* = 0.93) and decreased from 0.86 to 0 (*p* = 0.38) in the placebo group. OTU 068 (*Ruminococcus gnavus*), which is in a study by Azzouz et al. [9] was found to be highly represented in SLE patients, decreased from 38.96 to 17.65 (*p* = 1) in the synbiotic group and increased from 3.97 to 19.56 (*p* = 0.51) in the placebo group. 

From a Venn diagram (Figure 6b), six OTUs were identified as only being present after synbiotic supplementation. Five of these OTUs were *Firmicutes* (*Clostridia, Bacillus, Leuconostoc lactis, Streptococcus anginosus subsp. Whileyi, Veillonellaceae*), and one was an *Actinobacteria* (*Olsenella profusa*). These additional OTUs did not increase significantly with treatment. There were 13 OTUs in the synbiotic-supplemented group that disappeared after 60 days of synbiotic supplementation. Of these 13 OTUs, 9 were *Firmicutes* (*D. invisus, Ruminococcus albus, Peptoniphilus koenoeneniae, Lactobacillus fermentum, Gemella haemolysans, Clostridium spiroforme, Lachnoanaerobaculum, Leuoconstoc,* and *Oribacterium asaccharolyticum*), 2 were *Actinobacteria* (*Actinomyces odontolyticus* and *Gordonibacterium urolithinfacies*), 1 was *Cyanobacteria* (*Streptophyta*), and 1 was *Fusobacteria* (*Fusobacterium nucleatum subsp. Animalis*). Only 1 of these 13 OTUs, *D. invisus,* showed a marginally significant change (*p* = 0.08). Further details of the OTUs Venn diagram can be seen in a Supplementary File Table S3. All OTU changes can be seen in Figure 6c,d.

**Figure 7:**
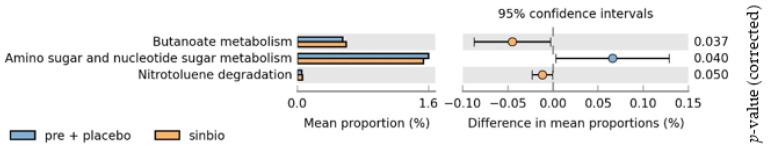
Changes in the functional prediction of gut microbiota after synbiotic supplementation.

To further understand the functional changes in the gut microbiome, we also performed functional predictions using the Phylogenetic Investigation of Communities by Reconstruction of Unobserved States (PICRUSt) bioinformatics package (http://picrust.github.io/picrust/ (accessed on 13 June 2018). Functional prediction revealed significant increases in butanoate or butyrate metabolism (*p* = 0.037) in the post-intervention synbiotic group compared with the pre-intervention synbiotic group and the placebo group (Figure 7). Amino sugar and nucleotide sugar metabolism significantly decreased in the post-intervention synbiotic group compared with the pre-intervention synbiotic group and placebo group (*p* = 0.040).

### 3.6. SLE Disease Activity

Statistical analysis using the Mann–Whitney test revealed that after 60 days of synbiotic supplementation, there was an improvement in SLE disease activity score measured by SLEDAI-2K. In synbiotic group median SLEDAI-2K were (14 [9; 16] vs. 8 [2; 12]; pre vs. post; *p* < 0.001). In placebo group, the median SLEDAI-2K were (9 [8; 18.25] vs. 9 [5.5; 15]; pre vs. post; *p* = 0.31). Using the Spearman rho correlation test, a trend of positive correlation was found between SLEDAI-2K score after intervention with hsCRP level (r = 0.24, *p* = 0.06), but not with IL-6 level (r = 0.17; *p* = 0.14). 

## 4. Discussion

A previous study showed that alterations in gut microbiota might be linked to SLE [2]. In the present study, we found increases in the proportions of *Bacteroidetes* and *Proteobacteria* and decreases in the proportion of *Firmicutes* and *Actinobacteria* in our patients with SLE compared with data of healthy subjects obtained from the literature. Our study only recruited SLE patients with gastrointestinal symptoms. In healthy subjects, the gut is inhabited by approximately 20% *Bacteroidetes*, 80% *Firmicutes*, 1% Proteobacteria, and 3% *Actinobacteria* [18]. In our study, the proportions of *Bacteroidetes, Firmicutes, Proteobacteria*, and *Actinobacteria* were 57%, 31.16%, 6.89%, and 0.64%, respectively, in the synbiotic group. In the placebo group, the proportions were 49.3%, 30.36%, 8.36%, and 0.59, respectively. A study from China reported increased *Bacteroidetes*, *Proteobacteria, Actinobacteria* and decreased Firmicutes in SLE patients. The present study lacks adequate data on healthy controls with similar genetic backgrounds. 

In this study, synbiotic supplementation was able to suppress the increase in hs-CRP levels seen in the placebo group. Serum hs-CRP levels are known to be higher in patients with SLE than in healthy controls [19] and are correlated with cardiovascular risk factors in patients with SLE [20]. One study showed an association of this measure with a disease activity score [21], but another suggested that such an association only appeared after excluding patients with measurable interferon (IFN)-α and *CRP* gene polymorphisms [22]. Our data are consistent with a reported meta-analysis showing the benefit of probiotic supplementation in reducing hs-CRP levels in other patient populations, such as those with rheumatoid arthritis [23]. To our knowledge, this was the first clinical trial using synbiotic preparation containing *Lactobacillus helveticus* R0052 60%, *Bifidobacterium infantis* R0033 20%, *Bifidobacterium bifidum* R0071 20%, and 80 mg fructo-oligosaccharides for SLE patients. In our study, there was also an improvement in SLE disease activity and the post-intervention SLEDAI-2K score showed a trend of positive correlation with hs-CRP level. Even though it was not statistically significant, the median baseline SLE disease activity in the synbiotic group was higher than in the placebo group. This can probably affect the result of the synbiotic supplementation. 

The effects of synbiotics observed in this study might be related to changes found in gut microbial composition and function. *Lactobacillus* supplementation in a classical animal model of lupus nephritis, MRL/lpr mice, decreased IL-6 levels in the gut [16]. IL-6 is a cytokine that controls CRP production by hepatocytes [24]. Another study revealed that *Lactobacillus rhamnosus* inhibits the formation of neutrophil extracellular traps [25], which increase IL-6 production from macrophages [26] and plasmacytoid dendritic cells [27]. We found an increase in the *Firmicutes/Bacteroidetes* ratio after synbiotic supplementation; however, this difference was not significant in comparison with placebo. Patients with SLE had lower *Firmicutes/Bacteroidetes* ratios compared with healthy controls [10], suggesting that an increased ratio might restore the balance of gut microbiota.

*Bacteriodetes* is the most abundant Gram-negative bacterial phylum in the gut microbiome and causes inflammatory conditions by secreting lipopolysaccharides and toxic proteolytic peptides [18]. *Firmicutes* is another dominant phylum in the gut microbiome [28], with butyrate as the predominant product. It is the source of energy for colonic epithelial cells and has anti-inflammatory effects [29]. In this study, *Bacteroidetes* among SLE patients with mild disease activity (SLEDAI-2K score <6) were significantly lower than patients with moderate or high disease activity (SLEDAI-2K score ≥6). Previous studies reported that SLE disease activity is positively correlated with *Ruminococcus gnavus, Streptococcus, Campylobacter, Streptococcus anginosus* and *Veillonella dispar* [8,10]. 

While in a previous study [7], *Ruminococcus gnavus* was found to be higher in patients with lupus nephritis, in our study it was not significantly different between SLE patients with or without renal involvement. We found that *Campylobacter* differed significantly between the two groups. 

In the synbiotic group, *B. salyersiae* and *D. invisus* decreased. *B. salyersiae* is an obligately anaerobic, Gram-negative bacterium that produces beta-lactamase and is resistant to penicillin G and vancomycin [30]. Yoon et al. isolated this bacterium from the peritoneal fluid of a patient with postoperative peritonitis [31]. Fecal *B. salyersiae* has been found to be inversely correlated to IFNγ production from peripheral blood mononuclear cells stimulated by *Candida albicans* [32]. *D. invisus* is an obligately anaerobic, Gram-negative coccobacillum that can be found in the normal gut and the dental root canals of patients with endodontic infections [33]. *Dialister* increases in the intestine of patients with Crohn’s disease [34] and is correlated with disease activity in patients with ankylosing spondylitis [35].

We identified six OTUs that were only found in the synbiotic group after intervention. Two of these OTUs, *Clostridia* and *Bacillus,* may have anti-inflammatory effects. Commensal *Clostridia* consists of Gram-positive, rod-shaped bacteria that can modulate immune responses and release butyrate, which inhibits the activation of the nuclear factor kappa-B, leading to decreased levels of proinflammatory cytokines [36]. Members of the *Bacillus* group are facultatively aerobic bacteria. Carbohydrates fermentation by the *Bacillus* species produce short-chain fatty acids, such as lactate, propionic, and butyrate, which affect host–microbe signaling, control colonic pH and regulate inflammation [37]. The four other OTUs, *L. lactis, S. anginosus subsp. whileyi, Veillonellaceae,* and *Olsenella profuse*, cause infections. *L. lactis* is a facultatively anaerobic Gram-positive coccus or coccobacillus that can cause infection in patients with risk factors such as compromised immunity [38]. *Veillonellaceae* can cause opportunistic infections which are usually polymicrobial but rarely severe monomicrobial infections [39]. *S.anginosus* is considered a commensal of the human gastrointestinal tract but can also cause opportunistic infections [40]. *O. profuse* is a Gram-positive, anaerobic bacterium isolated from subgingival plaques [41].

There were 13 OTUs in the synbiotic group before the intervention that were not found after the intervention. Eleven of these have the potential to be pathogenic: *D. invisus, L. fermentum, G. haemolysans, C. spiroforme, Lachnoanaerobaculum, Leuoconstoc, O. asaccharolyticum*, *A. odontolyticus, Streptophyta*, *F. nucleatum subsp. animalis,* and *P. koenoeneniae*. *L. fermentum* is a commensal bacterium that can modulate immune response but has also been reported as a pathogen in cholangitis of an 81-year-old male patient [42]. *G. haemolysans* is a Gram-positive coccus, facultatively anaerobic bacterium that can cause endocarditis, meningitis, endophthalmitis, liver abscesses, brain abscesses, and osteomyelitis, especially in immunocompromised patients [43]. There has also been a case report of peritonitis associated with *G. haemolysans* in immunocompetent patients [44]. *C. spiroforme*, which can cause enterocolitis and enterotoxaemia in rabbits, is not a cause of diarrhea in humans [45]. *Lachnoanaerobaculum* is a Gram-positive, rod-shaped, obligately anaerobic bacterium that has been isolated from the gut of a patient with celiac disease [46]. *Leuconostoc* is a Gram-positive coccus resistant to vancomycin that can cause infections in immunocompromised patients [47]. *O. asaccharolyticum* is an obligately anaerobic Gram-positive rod isolated from subgingival plaques [48]. *A. odontolyticus* is a Gram-positive, anaerobic bacterium colonizing the gut, respiratory tract, and female genital tract mucosa that can cause infection if the mucosal barrier is disrupted and also bacteremia in immunosuppressed patients [49]. *Streptophyta*, which is mostly found in the ocular microbiome, was also isolated from fecal specimens [50,51]. *F. nucleatum subsp. animalis* is an anaerobic Gram-negative bacterium mostly found in the oral cavity that can cause periodontal disease and is linked with inflammatory bowel disease and rheumatoid arthritis [52]. *P. koenoeneniae* is a Gram-positive, coccus-shaped, obligately anaerobic bacterium that was isolated from buttock abscesses in humans [53].

Two other OTUs found initially in the synbiotic group, *R. albus* and *G. urolithinfacies*, were not found after the intervention and are not pathogenic. *R. albus* is an anaerobic bacterium that contributes to fiber degradation [54]. *G. urolithinfaciens* is an obligately anaerobic Gram-positive coccobacillus that can metabolize ellagic acid from strawberries, walnuts and pomegranates to urolithin, which has anti-inflammatory effects [55]. In our study, *Ruminococcus gnavus* decreased in the synbiotic group and increased in the placebo group, but both changes were not statistically significant. A study by Azzouz et al. showed that patients with SLE had a five-fold greater representation of *R. gnavus* compared with control. As a result, SLE disease activity was correlated with *R. gnavus* relative abundance [12].

From functional predictions, we found significantly higher butyrate metabolism in the synbiotic group after treatment than in pre-intervention and in the placebo group. This result was in accordance with changes to the microbial composition showing increases in butyrate-producing bacteria. Butyrate has an anti-inflammatory effect and prevents nuclear factor kappa-B translocation to the nucleus, which decreases the transcription of genes encoding proinflammatory molecules such as IL-6 in peripheral blood mononuclear cells [56]. 

Nucleotide sugar and amino sugar metabolisms decreased in the synbiotic group after treatment. Nucleotide sugars are needed for glycoconjugate synthesis. Glycoconjugates form strain-specific barcodes on the surface of bacteria, mediating specific interactions with the host. Glycoconjugates such as glycoproteins, exopolysaccharides, capsular polysaccharides, lipopolysaccharides, lipo-oligosaccharides, lipoglycans, and peptidoglycans on the surface of pathogenic or beneficial bacteria can modify the host’s metabolism and immune system and are involved in the adhesion or evasion of bacteria. Changes in the levels of these glycoconjugates can affect the virulence of some pathogenic bacteria [57]. Amino sugars are utilized by bacteria for cell wall biosynthesis, and changes in their metabolism could decrease the expression of virulence factors [58]. 

This study has limitations. We only provided synbiotic supplementation for 60 days. With longer durations, alterations in microbial compositions might be more significant. Nevertheless, the strengths of the present study are the use of dietary assessments and the identification of medications that could affect the gut microbiome.

## 5. Conclusions

In conclusion, our data indicate that synbiotic supplementation for 60 days can alter the composition and function of the gut microbiome, dampen systemic inflammation, and improve SLE disease activity. Larger trials with longer duration of supplementation are needed to confirm the generalizability of the results.

## Figures and Tables

**Table 1:** Baseline characteristics.

Descriptive Characteristics	Synbiotic Treatment (*n* = 23)	Placebo (*n* = 23)	*p*
Age (y)	35 (24, 40)	27 (22, 40)	0.41
SLEDAI-2K score	14 (9, 18)	9 (8, 18)	0.32
Physician general assessment score	2 (1, 2)	1 (1, 2)	
Organ involvement (%)			
Lupus nephritis	10 (43)	10 (43)	1
Neuropsychiatric lupus	2 (9)	2 (9)	1
Hematology	2 (9)	7 (30)	0.14
Mucocutaneus	19 (56)	15 (44)	0.18
Musculosceletal	21 (91)	21 (91)	1
Antiphospholipid syndrome (%)	4 (17)	4 (17)	
Steroid dose per day (mg prednisone)	5 (5, 10)	5 (2.5, 7.5)	0.72
Immunosuppressant used (%)			
Hydroxychloroquine	3 (13)	2 (9)	1
Mycophenolate sodium	18 (78)	15 (65)	0.33
Azathioprine	2 (9)	5 (22)	0.41
Cyclosporine	0 (0)	2 (9)	0.5
>1 steroid sparing agent	2 (8.7)	1 (4.3)	
Vitamin D supplementation (%)	6 (26)	2 (9	0.24
Other medications (%)			
Proton pump inhibitors	10 (43)	11(48)	1
Statins	2 (9)	1(4)	0.77
BMI (kg/m^2^)	23.41 ± 6.19	22.09 ± 4.17	0.40
hs-CRP (mg/L)	2.4 (1, 5.9)	1.9 (0.4, 5.2)	0.48

Data are presented as mean ± standard deviation, n (%) or median (interquartile range). SLEDAI, Systemic Lupus Erythematosus Disease Activity Index; BMI, body mass index; hs-CRP, high sensitivity C-reactive protein.

**Table 2:** Daily intake from food sources.

Nutrient	Synbiotic Treatment (*n* = 21)				Placebo (*n* = 22)				*p*
	Pre	Post	*p*	Change Post-pre	Pre	Post	*p*	Change Post-pre	
Energy (kcal/day)	1318.54 ± 448.21	1024.8 ± 261.83	0.003	−294.27 ± 397.84	1352.37 ± 363.06	1081.40 ± 392.63	0.007	−270.97 ± 423.70	0.85
Fiber (g)	9.59 (6.70; 11.46)	5.57 (4.28; 8.50)	0.002	−3.85 (−5.03; −0.11)	8.58 (6.2; 13.88)	5.96 (4.33; 9.12)	0.005	−3.43 (−6.57; 0.43)	0.81
PUFA (g)	7.04 (4.43; 10.18)	4.21 (3.14; 6.41)	0.02	−2.35 ± 3.82	6.37 (5.01; 10.87)	5.43 (3.81; 6.60)	0.06	−2.48 ± 5.45	0.93
Vitamin A (μg)	835.98 (476.67; 1272.98)	576.96 (350.04; 1320.07)	0.46	−4.05 (−513.18; 177.95)	705.41 (516.82; 1001.18)	680.37 (399.91; 1120.00)	0.76	−30.14 (−358.16; 268.78)	0.81
Vitamin E (mg)	3.6 (2.42; 5.31)	2.4 (1.54; 2.93)	0.01	−0.62 (−2.62; 0.09)	3.21 (2.07; 4.14)	1.96 (1.37; 3.07)	0.01	−0.74 (−1.98; 0.01)	0.78
Vitamin C (mg)	67.06 (33.84; 103.43)	28.2 (21.49; 41.14)	0.001	−20.72 (−47.37; −4.09)	37.69 (16.46; 65.76)	27.63 (16.70; 41.03)	0.006	−8.47 (−31.38; 0.72)	0.25
Zinc (mg)	4.87 ± 1.77	3.81 ± 1.51	0.02	−1.06 ± 1.90	5.26 ± 2.27	4.02 ± 1.56	0.01	−1.25 ± 2.18	0.76

Intakes are presented as the mean ± standard deviation or median (interquartile range). PUFA, polyunsaturated fatty acid.

**Table 3:** Comparison of some variables according to disease activity and renal involvement.

	Disease Activity			Lupus Nephritis		
	SLEDAI-2K <6 (Mild Disease Activity)	SLEDAI-2K ≥6 (Moderate Disease Activity)	*p*	(+)	(−)	*p*
*Bacteroidetes*	43.19 ± 11.45	54.22 ± 13.81	0.04	51.91 ± 14.86	52.88 ± 13.74	0.87
*Firmicutes*	38.59 ± 13.88	30.48 ± 12.08	0.1	51.91 ± 14.86	52.88 ± 13.74	0.54
*Proteobacteria*	10.30 (4.54; 20.52)	7.05 (5.20; 12.78)	0.83	7.44 (4.92; 10.78)	6.89 (5.00; 17.89)	0.42
*Actinobacteria*	0.60 (0.37; 0.72)	0.52 (0.31;1.14)	0.79	0.53 (0.24; 1.05)	0.59 (0.38; 1.20)	0.68
*Firmicutes/Bacteroidetes* ratio	1.04 (0.56; 1.37)	0.52 (0.34; 0.72)	0.06	0.57 (0.39; 0.88)	0.54 (0.30; 1.04)	0.46
*Campylobacter* (OTU 149)	0 (0; 0)	0 (0; 0.01)	0.32	0 (0; 0.02)	0 (0; 0)	0.04
*Streptococcus* (OTU 062)	0.62 (0.24; 1.53)	0.14 (0.06; 0.97)	0.12	0.24 (0.08; 1.46)	0.13 (0.05; 0.77)	0.42
*Streptococcus anginosus* (OTU 241)	0 (0; 0)		0.92	0 (0; 0)		0.25
*Veillonella dispar* (OTU 059)	0.01 (0; 0.14)	0.01 (0; 0.11)	0.74	0.02 (0; 0.24)	0.01 (0; 0.05)	0.24
*Ruminococcus gnavus* (OTU 068)	0 (0; 0.01)	0.02 (0; 0.18)	0.13	0.04 (0; 0.28)	0.01 (0; 0.12)	0.13

Proportions are presented as the mean ± standard deviation or median (interquartile range). SLEDAI, Systemic Lupus Erythematosus Disease Activity Index.

## Data Availability

The metagenomics for the microbiome sequencing was uploaded to Sequence Read Archive (SRA) with accession number PRJNA761343.

## Supplementary Materials

The following supporting information can be downloaded at: https://www.mdpi.com/article/10.3390/cells11213419/s1, Table S1: Details of content, length, and number of sequences for each fecal sample; Table S2: Operational Taxonomic Units; Table S3: Venn-diagram details.
